# Minimal impacts on the wheat microbiome when *Trichoderma gamsii* T6085 is applied as a biocontrol agent to manage fusarium head blight disease

**DOI:** 10.3389/fmicb.2022.972016

**Published:** 2022-09-23

**Authors:** Arshani S. Alukumbura, Alessandro Bigi, Sabrina Sarrocco, W. G. Dilantha Fernando, Giovanni Vannacci, Marco Mazzoncini, Matthew G. Bakker

**Affiliations:** ^1^Department of Plant Science, University of Manitoba, Winnipeg, MB, Canada; ^2^Department of Agriculture, Food and Environment, University of Pisa, Pisa, Italy; ^3^Center of Agro-Environmental Research “Enrico Avanzi,” University of Pisa, Pisa, Italy; ^4^Department of Microbiology, University of Manitoba, Winnipeg, MB, Canada

**Keywords:** *Fusarium graminearum*, *Triticum durum*, *16S rRNA*, *ITS*, microbiome, fusarium head blight

## Abstract

Fusarium head blight (FHB) is a major fungal disease that causes severe yield and quality loss in wheat. Biological control can be integrated with other management strategies to control FHB. For this purpose, *Trichoderma gamsii* strain T6085 is a potential biocontrol agent to limit the infection of *F. graminearum* and *F. culmorum* in wheat. However, the possible impacts of *T. gamsii* T6085 on the broader microbiome associated with the wheat plant are not currently understood. Therefore, we identified bacteria and fungi associated with different wheat tissues, including assessment of their relative abundances and dynamics in response to the application of T6085 and over time, using amplicon sequencing. Residues of the prior year’s wheat crop and the current year’s wheat spikes were collected at multiple time points, and kernel samples were collected at harvest. DNA was extracted from the collected wheat tissues, and amplicon sequencing was performed to profile microbiomes using *16S v4 rRNA* amplicons for bacteria and *ITS2* amplicons for fungi. Quantitative PCR was performed to evaluate the absolute abundances of *F. graminearum* and *T. gamsii* in different wheat tissues. Disease progression was tracked visually during the growing season, revealing that FHB severity and incidence were significantly reduced when T6085 was applied to wheat spikes at anthesis. However, treatment with T6085 did not lessen the *F. graminearum* abundance in wheat spikes or kernels. There were substantial changes in *F. graminearum* abundance over time; in crop residue, pathogen abundance was highest at the initial time point and declined over time, while in wheat spikes, pathogen abundance increased significantly over time. The predominant bacterial taxa in wheat spikes and kernels were *Pseudomonas*, *Enterobacter*, and *Pantoea*, while *Alternaria* and *Fusarium* were the dominant fungal groups. Although the microbiome structure changed substantially over time, there were no community-scale rearrangements due to the T6085 treatment. The work suggests several other taxa that could be explored as potential biocontrol agents to integrate with T6085 treatment. However, the timing and the type of T6085 application need to be improved to give more advantages for T6085 to colonize and reduce the *F. graminearum* inoculum in the field.

## Graphical Abstract

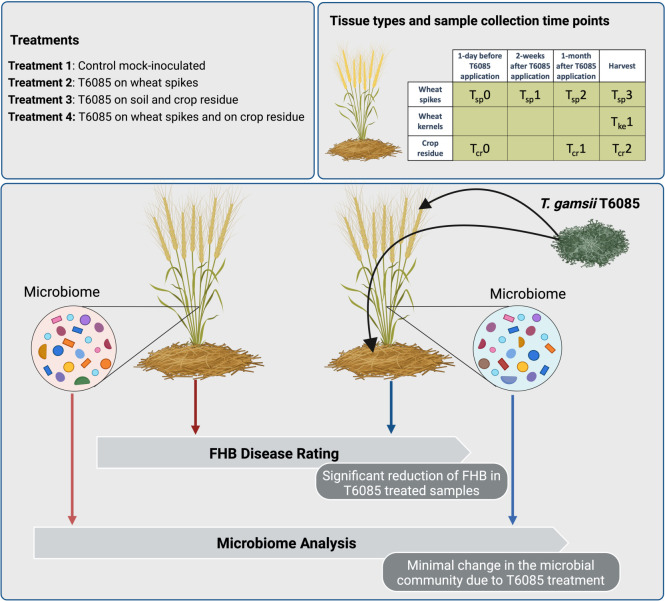

## Introduction

Fusarium head blight (FHB) is a prominent disease of wheat worldwide. The spread of the FHB pathogens reduces wheat production and causes toxins to contaminate harvested wheat grains, decreasing both the quality and quantity of wheat products (Gilbert and Tekauz, 2000; McMullen et al., 2012). *Fusarium* is the genus of pathogens that cause FHB, with primary causal agents being *F. graminearum* and *F. culmorum* (Walter et al., 2010). *Fusarium* infection typically leads to the premature bleaching of wheat spikes, although the timing of infection can alter the symptoms. The wheat anthers provide the perfect place for the *Fusarium* spp. to infect. Therefore, infections occur predominantly during the flowering period of the wheat plant. Earlier infections, such as during early anthesis, can lead to a failure to produce kernels. However, later infection gives rise to diseased wheat kernels with a shriveled and wilted appearance. In addition, *Fusarium* infections can also arise after the development of wheat kernels and the *Fusarium* infected wheat kernels are contaminated with toxins released by the fungus (Strange et al., 1974; Kang and Buchenauer, 2000; Birr et al., 2020).

*Fusarium graminearum* overwinters within crop residue. In this phase of its lifecycle, the pathogen acquires nutrients from decaying plant materials and lives saprotrophically during the fall, winter and spring seasons (Sutton, 1982). Different *Fusarium* spp. may have different environmental optima. Therefore, the abundance of pathogenic species or strains varies with the climatic conditions in different locations. Although *F. graminearum* can survive and develop within a wide range of temperatures (10–30°C), warm and humid environmental conditions favor spore production and successful infection (Osborne and Stein, 2007). Further, *F. graminearum* produces two types of spores. Ascospores are produced within perithecia, the reproductive structures of the sexual stage of the fungus. Ascospores are forcibly ejected from perithecia, which assists in these spores reaching turbulent air and traveling long distances via the wind. In contrast, the asexual spores, known as macroconidia, are not actively discharged into the air and travel shorter distances through rain splash (Fernando et al., 1997; Gilbert and Fernando, 2004). Upon the infection of wheat kernels, *Fusarium* releases mycotoxins. Type B trichothecenes are the major mycotoxins associated with FHB in wheat. Deoxynivalenol (DON) and nivalenol (NIV) are the major Type B trichothecenes. These mycotoxins affect the baking quality of wheat-based food products. They cannot be easily eliminated during food processing due to the stability of the toxins under typical food processing conditions. Consumption of mycotoxin-contaminated foods can lead to health problems in humans (Gärtner et al., 2008; Pestka, 2010), and therefore maximum tolerable mycotoxin levels are regulated in foods.

Within the strategies available to manage FHB and reduce the risk of mycotoxins entering the food chain, biological control by beneficial microorganisms such as bacteria, yeasts, and filamentous fungi, is receiving increasing attention due to pressure to reduce the use of pesticides and as an alternative to transgenic plants in those countries where their use is not allowed (Collinge and Sarrocco, 2022). Furthermore, other currently available tools for FHB management, such as agronomic practices, fungicides, and resistant wheat varieties, do not assure complete control of the disease (Sarrocco and Vannacci, 2018).

Among beneficial filamentous fungi that can be used in FHB management, those belonging to *Clonostachys* and *Trichoderma* are the most promising (Galletti et al., 2020; Gimeno et al., 2021). Among the mechanisms by which these fungi can antagonize *Fusarium* spp., competition for crop residue and space at the infection court (spikes) seems particularly important (Sarrocco et al., 2019a). The FHB pathogens overwinter and produce infectious spores on crop residue and find in spikes at flowering, the most susceptible phenological stage for host infection (McMullen et al., 2012).

In this context, *Trichoderma gamsii* strain T6085 has already been demonstrated to be able to reduce the growth of FHB pathogens (Matarese et al., 2012), as well as to control FHB symptoms under both lab and field conditions (Sarrocco et al., 2013; Vicente et al., 2020). In addition, T6085 can compete for natural substrates against the most important *Fusarium* species involved in FHB (Sarrocco et al., 2019b; Lasinio et al., 2021). The ability of T6085 to endophytically colonize wheat roots (Sarrocco et al., 2021) and spike tissues (Sarrocco et al., 2013), combined with its ability to modulate the expression of defense-related genes in the host (Vicente et al., 2020), render this isolate an excellent candidate to be developed as the active ingredient of biopesticides to be used both in organic and integrated pest management strategies (Jensen et al., 2016).

However, pathogens such as *Fusarium* spp. are members of more complex microbiomes, including numerous other fungi and bacteria, that also live in association with different host tissues. These microbiomes are frequently divided among rhizosphere, phyllosphere, and endosphere communities (Turner et al., 2013), and extend to the residue that remains after a host plant has died (Bakker et al., 2017). Many beneficial functions are provided by the plant-associated microbiome, including facilitated nutrient uptake, promotion of plant growth and development, and induction of host plant resistance toward pathogens (Smith and Goodman, 1999). Therefore, as for other disease management strategies, it is relevant to assess how biological control approaches impact microbiomes to control pathogens. Current studies on the dynamics of microbiomes associated with various crops in response to the application of biocontrol agents have shown different impacts, but these are typically either transient (Chowdhury et al., 2013; Yin et al., 2013) or a minimal impact that did not create a measurable change in the microbial communities over time (Scherwinski et al., 2007; Perazzolli et al., 2014; Wei et al., 2016). This is the first study looking for a variation in wheat associated microbial community under the treatment of *T. gamsii* T6085 as a biocontrol agent to mitigate FHB in wheat. During this study we investigated the changes in microbial communities over time and incorporated different methods of introducing the biocontrol agent to compare and contrast their effectiveness.

This study aimed to evaluate the impact of T6085 on the natural fungal and bacterial communities that interact with various wheat tissues. It is crucial to determine the non-target effects on the microbiome due to the artificial inoculation of a biocontrol agent, as these could affect both plant and soil health. Therefore, the microbial communities associated with different wheat tissues were profiled, and their dynamics were observed at different time points in response to different T6085 treatments.

## Materials and methods

### Fungal material and inoculum preparation

*Trichoderma gamsii* strain T6085 has been studied for several years (Matarese et al., 2012). This strain was originally isolated from an uncultivated soil in Crimea (Ukraine), and its genome was sequenced and annotated (Baroncelli et al., 2016). Strain T6085 is deposited at the Fungal Collection of the Plant Pathology Lab (University of Pisa, Italy).

Inoculum of strain T6085 was prepared for application in the field trial using two different methods. For inoculation of wheat spikes, an aqueous suspension of spores (10^6^ spores mL^–1^) was prepared on the day of application from cultures on potato dextrose agar (PDA) plates (24°C, 12 h light/12 h darkness for 2 weeks). For inoculation of crop residues from the prior cropping season, inoculum was prepared on whole millet grain. Briefly, 15 mL of aqueous spore suspension (10^5^ spores mL^–1^, produced by cultivation on PDA as above) was used to inoculate 1 kg of millet seeds in sterile plastic bags (previously wet with 300 mL of distilled water and sterilized by autoclaving for 20 min at 121°C). The millet seeds were incubated at 24°C under 12 h light/12 h darkness for 10 days, then dried in a laminar flow hood and stored at 4°C until use.

### Field experiment

#### Experimental design

An experiment was performed during the 2018–2019 cropping season to evaluate the effect of the application of T6085 on bacterial and fungal communities naturally associated with wheat residue, spikes and kernels. The experiment was established at the Center of Agro-environmental Research “Enrico Avanzi,” San Piero a Grado, Pisa (University of Pisa, Italy). Durum wheat (*Triticum durum* cv *Tirex*) was sown on 11 December 2018 in a no-tillage system, using a no-till seeder and applying about 400 seeds m^–2^ in rows 15 cm apart from each other. P and K mineral fertilizers were applied before sowing (30 and 107 kg ha^–1^, respectively); mineral nitrogen was applied splitting the total amount (170 kg ha^–1^) equally between the stages of beginning stem elongation and tillering. Weeds were controlled using a post-emergence herbicide application (Atlantis flex, a.i.: propoxycarbazone-sodium + mesosulfuron-methyl). The crop was harvested on the 24 July 2019.

Plots were assigned using a randomized block design (randomization via Random.org) with five blocks and a plot size of 6 m wide × 9 m long (40 rows for plot). Four treatments were implemented: (1) Non-inoculated control; (2) wheat spikes at anthesis inoculated with T6085 (i.e., to place T6085 in competition with *Fusarium* at the site of infection); (3) crop residues from the previous cropping season (which was also of wheat) inoculated with T6085 (i.e., to create competition between T6085 and *Fusarium* at the site of spore production); (4) both spikes at anthesis and crop residues inoculated with T6085 (i.e., to combine biocontrol effects at both the site of inoculum production and at the site of infection). Natural *Fusarium* inoculum was relied upon for the development of FHB.

#### Inoculation and sampling of crop residues

At the beginning of stem extension (Feekes stage 6), T6085 was introduced to the soil surface of appropriate plots (i.e., Treatments 3 and 4) by distributing colonized millet seeds by hand (4 g m^–2^) while walking linearly into the field and spreading the inoculum all over the plot. For treatments with no inoculation of crop residues (i.e., Treatments 1 and 2), sterile millet seeds were distributed in the same manner and at the same rate. Samples of residue from the prior cropping season were collected 1 day before the introduction of T6085 (denoted as T_*cr*_0, for “time 0, crop residue”), after 1 month (T_*cr*_1), and just before the harvest (T_*cr*_2). At each sample collection, a quadrat was marked by placing a 50 cm × 50 cm frame within the central region (2 × 3 m) of each plot and 50 pieces of wheat straw residue were collected by hand from within the quadrat (quadrat method). Residue pieces were cut longitudinally, washed three times for 1 min in sterile water to remove soil particles, dried in a sterile laminar flow hood, ground with a Cyclotech 1093 Sample mill and stored at –20°C until use for microbiome profiling.

#### Inoculation and sampling of wheat spikes

At anthesis, T6085 inoculum was applied on spikes (120 L ha^–1^) for treatments 2 and 4, while treatments 1 and 3 were given an equal volume of sterile water as a mock inoculum. Spikes were sampled using the quadrat method described above, taking a subsample of 10 spikes per sample. Spike sample collection took place 1 day before the introduction of T6085 to spikes (denoted as T_*sp*_0, for “time 0, spike tissue”), 14 days post-inoculation (dpi) (T_*sp*_1), 28 dpi (T_*sp*_2), and at harvest (35 dpi; T_*sp*_3). From each spike, 6 spikelets were subsampled (two from the third lower, two from the middle and two from the upper third of the spike) were collected and stored at –20°C until use for microbiome profiling.

#### Assessment of fusarium head blight severity and agronomic parameters

At time points T_*sp*_1, T_*sp*_2 and T_*sp*_3, an additional 30 spikes from each plot were observed for FHB symptoms. Disease Incidence (DI) was defined as the % of spikes showing premature bleaching of some or all spikelets. Disease Severity (DS) was defined as the % of spikelets within a spike that were symptomatic. At the end of the cropping season, wheat was harvested, and several agronomical parameters were evaluated on sub-samples, such as spike number (count m^–2^), straw and spike fresh weight (g m^–2^) and total kernel weight (g m^–2^).

The DI and DS values, after angular transformation, and agronomical data were submitted to analysis of variance (ANOVA) and Tukey’s *post hoc* contrasts by using SygmaPlot 12.0 to evaluate the effect of T6085 application on disease development and crop productivity. The threshold for statistically significant effects was set at *p* ≤ 0.05.

### Amplicon sequencing

Ground tissue samples were stored in DNA/RNA Shield (ZymoResearch) and shipped by express courier to Winnipeg, Canada. The DNA was extracted from a 0.1 g subsample using the GeneJet plant genomic DNA extraction kit (Thermo Fisher Scientific). The concentration of extracted DNA was diluted to a consistent 2 ng μL^–1^, based on spectrophotometric concentration measurement (NanoDrop One, Thermo Fisher Scientific). The *ITS2* region of the fungi (employing ITS3_KYO2 forward primer and ITS4_KYO3 reverse primer) and the v4 region of *16S rRNA* gene of bacteria (employing F515 forward primer and R806 reverse primer) were amplified (Caporaso et al., 2011; Toju et al., 2012). Primers were modified with 5′ overhangs for compatibility with later indexing steps and included variable length spacers to increase the signal diversity when sequencing through the primer region. The 20 μL PCR mixtures consisted of KAPA HiFi HotStart master mix (Roche), forward and reverse primers at 0.5 μM each, nuclease-free water, and template DNA at a final concentration of 0.8 ng μL^–1^. To inhibit the amplification of mitochondrial and chloroplast DNA from the host plant, mPNA and pPNA blockers (PNAbio) were included at 0.25 μM in the *16S rRNA* amplification only (Table 1).

**TABLE 1:** The PCR conditions used for amplicon generation, indexing, and quantitative PCR.

Reaction	Primers	PCR program
Amplicon PCR	*ITS2*	Initial denaturation at 98°C for 3 min, 25 cycles of (denaturation at 98°C for 15 s, annealing 57°C for 10 s, extension 72°C for 1 min), final extension 72°C for 10 min
	*16S rRNA*	Initial denaturation at 98°C for 3 min, 25 cycles of (denaturation at 98°C for 15 s, annealing 57°C for 10 s, PNA clamping 75°C 10 s, extension 72°C for 1 min), final extension 72°C for 10 min
Index PCR	Nextera XT index kit	Initial denaturation at 98°C for 3 min, 8 cycles of (denaturation at 98°C for 30 s, annealing 55°C for 30 s, extension 72°C for 30 s), final extension 72°C for 5 min
qPCR	Fus.GRA	Initial denaturation at 98°C for 3 min, 40 cycles of (denaturation at 98°C for 15 s, annealing/extension at 58°C for 30 s, plate read), melt curve analysis up to 65–95°C at 3 s/increment
	Tgam	Initial denaturation at 98°C for 3 min, 40 cycles of (denaturation at 98°C for 15 s, annealing/extension at 56°C for 30 s, plate read), melt curve analysis up to 65–95°C at 3 s/increment

Amplified PCR products were purified using KAPA HyperPure beads (Roche). Dual indices and adapters were attached via the Nextera XT Index Kit (Illumina), according to the manufacturer’s protocol. Indexed samples were pooled in equimolar quantities. Because some smaller than expected fragments (likely primer dimers) were present in the library, the pooled library was size-selected using a Flashgel recovery cassette (Lonza) and purified again with KAPA HyperPure beads. The amplicon size distribution of the pooled library was visualized using the TapeStation system (Agilent), and the concentration of MiSeq-compatible amplicons was determined using the KAPA Library Quantification Kit for Illumina Platforms (Roche). PhiX was spiked into each library at a rate of 7.5%. The samples were split across four sequencing runs. The MiSeq v2 reagent kit (2 × 250 bp) was used for v4 *16s rRNA* libraries, and the v3 reagent kit (2 × 300 bp) was used for *ITS2* libraries. Negative controls (PCR reactions without template DNA) and mock communities were included in each sequencing run. For the bacterial libraries, the mock community was product MSA-3002 from the American Type Culture Collection, and for the fungal libraries, a mock community from Bakker (2018) was used. Unprocessed sequence data are available as accession PRJNA790613 at the NCBI SRA.

The sequence data were processed, and taxonomic identifications were assigned using CLC Genomics Workbench version 20.0.4 (Qiagen). The low-quality sequences (limit 0.05) and sequence ends containing ambiguous nucleotides (maximum 2) were trimmed off. Adapter sequences were trimmed and reads below a length of 15 were discarded. Reference-based operational taxonomic unit (OTU) clustering was performed at a 99% similarity percentage. To identify the likely taxonomic origin of fungal amplicons, the UNITE database v7.2 (99% with singletons, developer version) was used (Nilsson et al., 2018), while the SILVA 16S v132 99% database (Quast et al., 2013) was used for bacterial amplicons. Processed sequence variant lists, tables of abundances across samples, taxonomic affiliations, and metadata were stored as phyloseq objects in R (McMurdie and Holmes, 2013). Indices of diversity, including OTU richness, Shannon alpha diversity index, and Bray-Curtis pairwise dissimilarities, were calculated using the package vegan (Oksanen et al., 2022). Testing for community-scale differences among treatments via Adonis also used the package vegan. Box plots and ordinations were created using the package ggplot2 in R (Wickham, 2016). Differential expression analyses were performed using DEseq2 (Love et al., 2014). In testing for the differential relative abundance of OTUs, treatments were grouped according to whether or not T6085 was applied to the tissues in question; for example, with respect to kernel samples, the presence of T6085 was equivalent across Treatment 1 (non-inoculated control) and Treatment 3 (T6085 applied to crop residue only). OTU abundances were modeled on treatment, time point, the interaction between treatment and time point, and the blocking factor in the experimental design.

### Quantitative PCR

Quantitative PCR (qPCR) was performed to calculate the densities of *T. gamsii* and *F. graminearum* within samples, using assays *Fus.GRA* and *Tgam* from Sarrocco et al. (2019b; Table 1). The qPCR mixture consisted of 10 μL of 2 × SYBR Green Master Mixture (Bio-Rad), forward and reverse primers at 0.5 μM each, nuclease-free water, and template DNA. Efficiencies when amplifying genomic DNA of the target organisms were 94.06% for the *Fusa.GRA* assay and 92.95% for the *Tgam* assay. The abundances of *T. gamsii* and *F. graminearum* were calculated using the standard curve method. Changes in *F. graminearum* densities over time and due to the presence of *T. gamsii* T6085 were analyzed by ANOVA, modeling log-transformed abundance values on treatment, time point, the interaction between treatment and time point, and the blocking factor in the experimental design. The Tukey HSD method was used to identify significant contrasts where the main effects were significant.

## Results

### Fusarium head blight development and severity in the field

**FIGURE 1:**
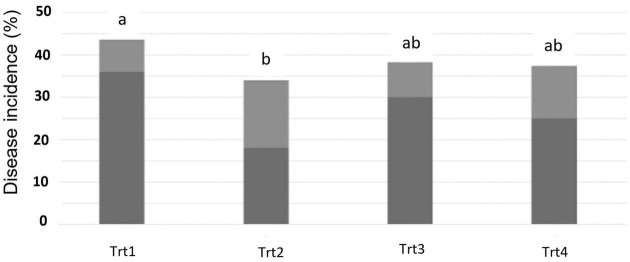
Fusarium head blight disease incidence, as assessed at 28 days post inoculation of T6085. Data are the mean (dark gray) + SD (soft gray) percentage of spikes showing visual symptoms of FHB (*n* = 5 replicates, with 30 spikes assessed per sample). Trt1 was the non-inoculated control. In Trt2, *T. gamsii* strain T6085 spore suspension was applied onto wheat spikes at anthesis. In Trt3, T6085 was applied to crop residue on the soil surface. In Trt4, T6085 was applied onto both spikes and crop residue at anthesis. Treatment means indicated by different letters are significantly different (ANOVA with Tukey HSD, *p* < 0.05).

At 14 dpi of T6085, there was no naturally occurring disease in any spikes collected. However, by 28 dpi of T6085, the typical symptoms of FHB blight, such as bleaching of some or all the spikelets within each spike, appeared. A reduction of DI was recorded in all the treatments in which T6085 was applied as spike and/or soil inoculum, with Treatment 2 (T6085 applied to flowering spikes) showing a significantly reduced percentage of affected spikes (Figure 1, *p* ≤ 0.05).

**FIGURE 2:**
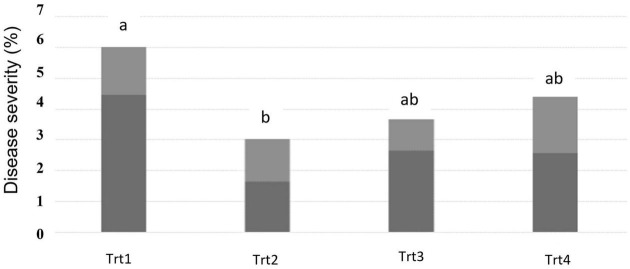
Fusarium head blight disease severity as assessed at 28 days post inoculation of T6085. Data are the mean (dark gray) + SD (soft gray) percentage of spikelets within a spike showing visual symptoms of FHB (*n* = 5 replicates, at 30 spikes assessed per sample). Trt1 was the non-inoculated control. In Trt2, *T. gamsii* strain T6085 spore suspension was applied to wheat spikes at anthesis. In Trt3, T6085 was applied to crop residue at the soil surface. In Trt4, T6085 was applied to both spikes at anthesis and crop residue. Treatment means indicated by different letters are significantly different (ANOVA with Tukey HSD, *p* < 0.05).

The pattern of effects for DS was the same as was observed for DI; the percentage of affected spikelets within each spike was generally reduced in all the plots where T6085 was applied, but the decrease was only statistically significant in the contrast of Treatment 2 vs. the non-inoculated control (Figure 2, *p* ≤ 0.05).

By 35 dpi of T6085, differences among treatments in DI and severity were no longer apparent (data not shown), thus demonstrating that the beneficial effect of the antagonist did not last over a long time.

### Agronomic parameters

At the end of the cropping season (on 24 July 2019), wheat was harvested from all plots, and agronomic factors were measured to evaluate the possible effects of the application of T6085 on wheat yield and yield components. There were no significant differences among treatments in spike density or the biomass of straw, spikes, or grain at harvest (Table 2; ANOVA with Tukey HSD, *p* > 0.05).

**TABLE 2:** Effect of treatments on some agronomic traits of wheat.

Treatment	Spike density (count m^–2^)	Straw fresh weight (g m^–2^)	Spike fresh weight (g m^–2^)	Grain yield (g m^–2^)
**Treatment 1:** Non-inoculated control	285 a ± 53.4	249 a ± 45.6	342 a ± 74.9	225 a ± 82.3
**Treatment 2:** T6085 to spikes	302 a ± 49.6	259 a ± 33.7	361 a ± 73.5	248 a ± 60.5
**Treatment 3:** T6085 to crop residue	295 a ± 81.2	245 a ± 49.4	317 a ± 75.4	202 a ± 70.1
**Treatment 4:** T6085 to spikes + crop residue	309 a ± 45.3	250 a ± 31.7	341 a ± 57.1	231 a ± 42.2

Values are means followed by a standard deviation of 5 plot-level replicates. Shared lowercase letters represent no statistically significant difference between treatments (ANOVA with Tukey HSD, *p* > 0.05).

### The abundance of *Fusarium graminearum* in wheat tissues

**FIGURE 3:**
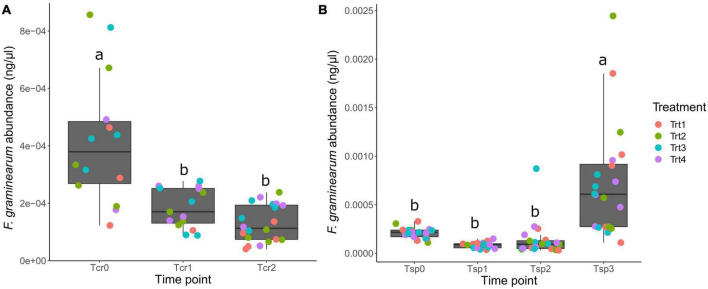
Abundance of *F. graminearum* over time in **(A)** crop residue and **(B)** wheat spikes. See the text for a complete description of the sampling time points (T_*cr*_0, T_*cr*_1, T_*cr*_2; T_*sp*_0, T_*sp*_1, T_*sp*_2, T_*sp*_3). Individual data points are colored by treatment, where Trt1 (non-inoculated control), Trt2 (T6085 applied to spikes at anthesis), Trt3 (T6085 applied to crop residue) and Trt4 (T6085 applied to both spikes and to crop residue). Box plots indicate the 1st quartile, median and 3rd quartile. Different letters within each panel indicate significant differences between the time points (log-transformed *F. graminearum* abundances modeled on treatment, time point, the interaction between treatment and time point, and the blocking factor in the experimental design; Tukey HSD contrasts among time points, *p* ≤ 0.05).

The abundance of *F. graminearum* changed over time in crop residues and wheat spikes. In crop residues, *F. graminearum* abundance ranged from 4.03 × 10^–5^ to 8.57 × 10^–5^ ng μL^–1^ of DNA, while there was an extensive range in abundances in wheat spikes, from 3.11 × 10^–5^ to 2.44 × 10^–3^ ng μL^–1^ of DNA. In crop residues, the *F. graminearum* density was significantly higher at the initial time point (T_*cr*_0), and it decreased over time (ANOVA with Tukey HSD, *p* ≤ 0.05). The abundance of *F. graminearum* in wheat spikes showed an opposite trend, as values were significantly higher at the last time point (T_*sp*_3) compared to all earlier time points (Figure 3; ANOVA with Tukey HSD, *p* ≤ 0.05).

**FIGURE 4:**
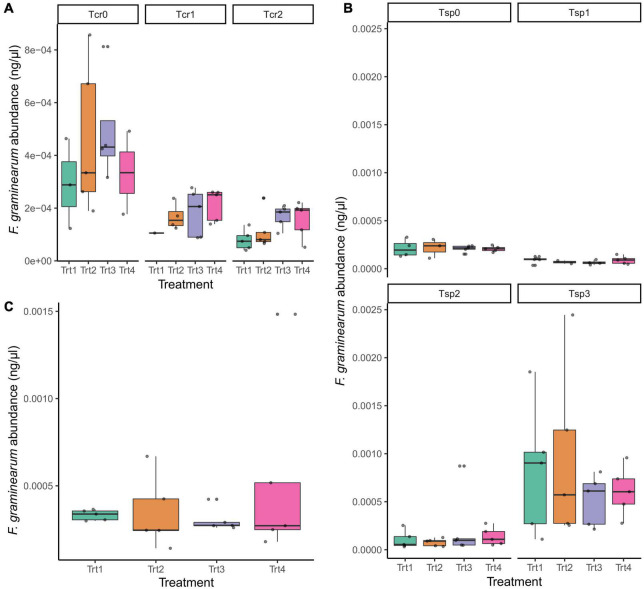
The impact of T6085 application on *F. graminearum* abundance, as measured by quantitative PCR, in **(A)** crop residue; **(B)** wheat spikes; **(C)** wheat kernels. See text for a complete description of the sampling time points (T_*cr*_0, T_*cr*_1, T_*cr*_2; T_*sp*_0, T_*sp*_1, T_*sp*_2, T_*sp*_3). The *x*-axis labels indicate the experimental treatments: Trt1 (non-inoculated control), Trt2 (T6085 applied to spikes at anthesis), Trt3 (T6085 applied to crop residue), Trt4 (T6085 applied to both spikes and to crop residue). Box plots indicate the 1st quartile, median and 3rd quartile. Within panels, there were no statistically significant differences among treatments (log-transformed *F. graminearum* abundances modeled on treatment, time point, the interaction between treatment and time point, and the blocking factor in the experimental design; *p* > 0.05 for treatment factor in all cases).

Across experimental treatments, there were no significant changes in *F. graminearum* abundance due to the application of T6085 (Figure 4; ANOVA with Tukey HSD, *p* > 0.05).

### Variation in microbial diversity

The number of observed OTUs (i.e., richness) and Shannon diversity index were calculated to further understand the dynamics in the microbial community in different wheat tissues due to the T6085 treatment and over time. Crop residue had bacterial and fungal richness within the range between 1,343 and 2,889 OTUs and 74–178 OTUs, respectively. In both wheat spikes and kernels, fungal richness was less than 100 OTUs, while bacterial OTU richness was comparatively higher. The richness values did not change significantly in response to the application of T6085 (Table 3). However, there was a significant variation in the richness over time in bacterial and fungal communities residing in crop residue and wheat spikes (ANOVA with Tukey HSD, *p* ≤ 0.05). In crop residue, bacterial richness increased significantly from T_*cr*_1 to T_*cr*_2, and fungal richness increased significantly over time. In wheat spikes, bacterial richness was significantly lower at T_*cr*_0 than at other time points, while the fungal richness significantly decreased from T_*sp*_1 to T_*sp*_2 (Table 4).

**TABLE 3:** The microbial richness (number of OTUs) and Shannon index of diversity for bacteria and fungi in crop residue, wheat spikes and kernel tissues collected at different time points under different T6085 treatments.

Type of tissue	Time point	Treatment*	Bacteria		Fungi	
			OTU richness	Shannon index	OTU richness	Shannon index
Residue	T_*cr*_0	Untreated	1857.7 ± 222	5.8 ± 0	93.3 ± 12	2.5 ± 0
Residue	T_*cr*_1	Treated	2037.7 ± 245 a	5.9 ± 0 a	118.2 ± 18 a	2.8 ± 0 a
		Untreated	1922.8 ± 332 a	5.8 ± 0 a	109.2 ± 25 a	2.7 ± 0 a
Residue	T_*cr*_2	Treated	2148.9 ± 360 a	6.1 ± 0 a	129.2 ± 19 a	3.0 ± 0 a
		Untreated	1888.1 ± 209 a	6.0 ± 0 a	126.7 ± 28 a	3.0 ± 0 a
Spikes	T_*sp*_0	Untreated	14.4 ± 5	1.0 ± 0	5.6 ± 2	1.5 ± 0
Spikes	T_*sp*_1	Treated	35.6 ± 6 a	2.0 ± 0 a	6.6 ± 1 a	1.7 ± 0 a
		Untreated	28.9 ± 4 a	1.8 ± 0 a	5.8 ± 1 a	1.6 ± 0 a
Spikes	T_*sp*_2	Treated	32.7 ± 8 a	1.8 ± 0 a	4.4 ± 2 a	1.1 ± 1 a
		Untreated	32.5 ± 7 a	1.8 ± 0 a	4.5 ± 1 a	1.2 ± 0 a
Spikes	T_*sp*_3	Treated	32.6 ± 6 a	2.1 ± 0 a	5.1 ± 1 a	1.5 ± 0 a
		Untreated	38.0 ± 6 a	2.3 ± 0 a	5.2 ± 1 a	1.5 ± 0 a
Kernels	T_*ke*_1	Treated	206.4 ± 61 a	1.7 ± 1 a	55.7 ± 4 a	2.0 ± 0 a
		Untreated	229.3 ± 70 a	1.9 ± 1 a	52.0 ± 6 a	2.0 ± 0 a

Values shown are means ± standard deviation. There were no significant impacts of treatment with T6085 on these community characteristics (ANOVA with Tukey HSD, *p* > 0.05).

*Treatments were grouped according to whether or not T6085 was applied to the tissues in question; for example, with respect to kernel samples, the presence of T6085 was equivalent across Treatment 1 (non-inoculated control) and Treatment 3 (T6085 applied to crop residue only); therefore, these treatments were combined as “Untreated”.

**TABLE 4:** The microbial richness (number of OTUs) and Shannon index of diversity for bacteria and fungi in crop residue, wheat spikes and kernel tissues collected at different time points.

Type of tissue	Time point	Bacteria		Fungi	
		OTU richness	Shannon index	OTU richness	Shannon index
Residue	T_*cr*_0	1857.7 ± 222 b*	5.8 ± 0 b	93.3 ± 12 c	2.5 ± 0 b
	T_*cr*_1	1999.4 ± 270 b	5.9 ± 0 b	115.2 ± 20 b	2.8 ± 0 b
	T_*cr*_2	2018.5 ± 316 a	6.1 ± 0 a	128.0 ± 23 a	3.0 ± 0 a
Spikes	T_*sp*_0	14.4 ± 5 b	1.1 ± 0 b	5.6 ± 2 ab	1.5 ± 1 ab
	T_*sp*_1	31.8 ± 6 a	1.9 ± 0 a	6.1 ± 1 a	1.7 ± 0 a
	T_*sp*_2	32.6 ± 7 a	1.8 ± 0 a	4.5 ± 2 b	1.2 ± 1 b
	T_*sp*_3	35.3 ± 7 a	2.2 ± 0 a	5.2 ± 1 ab	1.5 ± 0 ab
Kernels	T_*ke*_1	217.8 ± 65	1.8 ± 1	53.9 ± 6	2.0 ± 0

Values shown are means ± standard deviation.

*Among time points within tissue types, values followed by different lower-case letters were significantly different from each other (ANOVA with Tukey HSD, *p* < 0.05).

Similarly, the Shannon index of bacterial and fungal OTU diversity was not significantly changed by the application of T6085 (Table 3). However, there was a significant variation in the Shannon index over time in microbes associated with wheat spikes and crop residue (Table 4). The Shannon index of both bacteria and fungi increased over time, reaching a significantly higher value at T_*cr*_2 compared to T_*cr*_0 (ANOVA with Tukey HSD, *p* ≤ 0.05). Further, bacterial diversity was significantly higher at all later time points compared to T_*sp*_0 in wheat spikes, while there was a significant increase in diversity in the fungal community associated with wheat spikes from T_*sp*_1 to T_*sp*_2 (ANOVA with Tukey HSD, *p* ≤ 0.05).

### Variations in the microbial community due to treatment and over time

**FIGURE 5:**
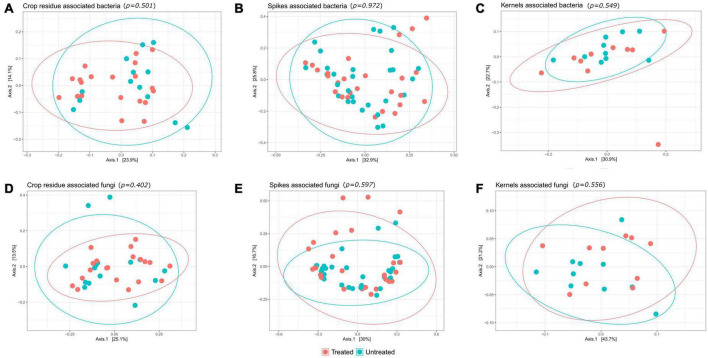
Application of T6085 causes no broad changes in microbiomes of wheat. Plots are principal coordinates ordinations derived from pairwise community dissimilarity values (Bray-Curtis index). Panels represent bacterial communities from **(A)** crop residue; **(B)** wheat spikes; **(C)** wheat kernels; and fungal communities from **(D)** crop residue; **(E)** wheat spikes; **(F)** wheat kernels. Treatments have been grouped according to whether or not *T. gamsii* T6085 was applied to the tissue in question. Ellipses represent a 95% confidence level. The fungal and bacterial microbiomes did not show any significant community scale change due to T6085 treatment (Adonis test, *p-values* shown above each panel).

The changes in the microbial community due to the T6085 treatment were assessed using Bray-Curtis dissimilarities with principal coordinate analysis. Neither bacterial nor fungal community structures were appreciably changed due to the T6085 treatment (Adonis test, *p* > 0.05) (Figure 5).

Even though there were no whole community rearrangements in response to the application of T6085, some individual taxa showed differential abundance due to the treatments in wheat spikes samples (DEseq, *p* ≤ 0.05) (Supplementary Table 1). These differentially abundant taxa included members of *Massilla, Fusarium* and *Alternaria*. *Massilla* showed a significantly higher abundance in T6085 treated samples collected at T_*sp*_3, *Alternaria* showed a significantly lower abundance in T6085 treated wheat spikes samples collected at T_*sp*_1, and a *Fusarium* sequence variant showed a reduced abundance in treated samples at T_*sp*_2 while another *Fusarium* sequence variant showed a significantly higher abundance in T6085 treated samples collected at T_*sp*_2.

We explored network analyses (i.e., patterns of association among sequence variants across samples) as an alternative approach to reveal whether broad community rearrangements could be observed. Interestingly, for microbiomes associated with wheat spikes, we observed only half as many significant associations among sequence variants for samples that had been treated with T6085, compared to samples that had not been treated with T6085 (Supplementary Figure 1). For example, edge density was 0.0193 for the network from untreated samples and was 0.00955 for the network from treated samples; similarly, the mean number of significant associations per sequence variant (i.e., mean degree per node) was double (3.06 vs. 1.51) for the network from untreated samples, compared to the network from treated samples (Supplementary Figure 1). However, at the scale of the network, we have only *n* = 1 observations, and so these comparisons must be taken with a grain of salt.

**FIGURE 6:**
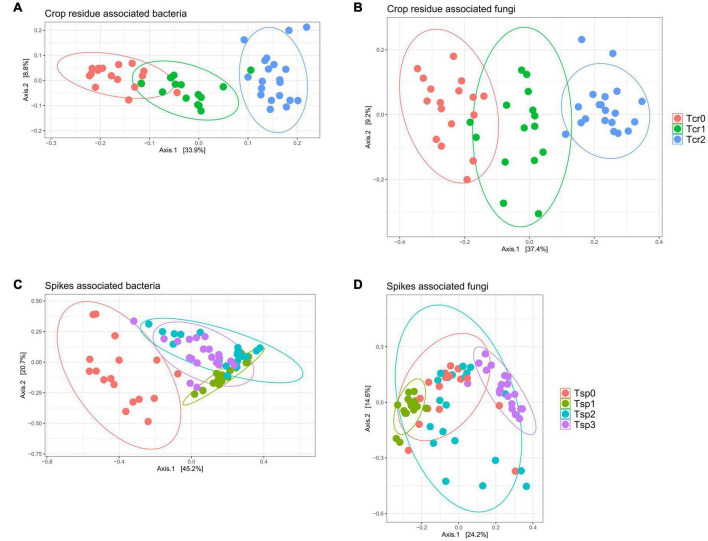
Microbiomes associated with wheat tissue demonstrate progressive change over time. Plots are principal coordinates ordinations derived from pairwise community dissimilarity values (Bray-Curtis index). **(A)** Bacterial communities from crop residue; **(B)** fungal communities from crop residue; **(C)** bacterial communities from wheat spikes; **(D)** fungal communities from wheat spikes. See text for a complete description of the sampling time points (T_*cr*_0, T_*cr*_1, T_*cr*_2; T_*sp*_0, T_*sp*_1, T_*sp*_2, T_*sp*_3). Ellipses represent a 95% confidence level.

Despite the minimal variations in fungal and bacterial community structures due to the application of T6085, there were apparent changes in fungal and bacterial community composition over time in crop residue and wheat spikes (Figure 6). In crop residue samples, differentially abundant OTUs numbered 446 for the contrast between T_*cr*_0 and T_*cr*_1, 1 040 between T_*cr*_0 and T_*cr*_2, and 589 between T_*cr*_1 and T_*cr*_2. In wheat spike samples, differentially abundant OTUs numbered 76 between T_*sp*_0 and T_*sp*_1, 120 between T_*sp*_0 and T_*sp*_2, 111 between T_*sp*_0 and T_*sp*_3, 44 between T_*sp*_1 and T_*sp*_2, 68 between T_*sp*1_ and T_*sp*_3 and 69 between T_*sp*_2 and T_*sp*_3 (Supplementary Tables 2, 3). Thus, many microbial taxa associated with wheat demonstrated significant shifts in relative abundance over weeks.

### The composition of microbiomes associated with wheat tissues

**FIGURE 7:**
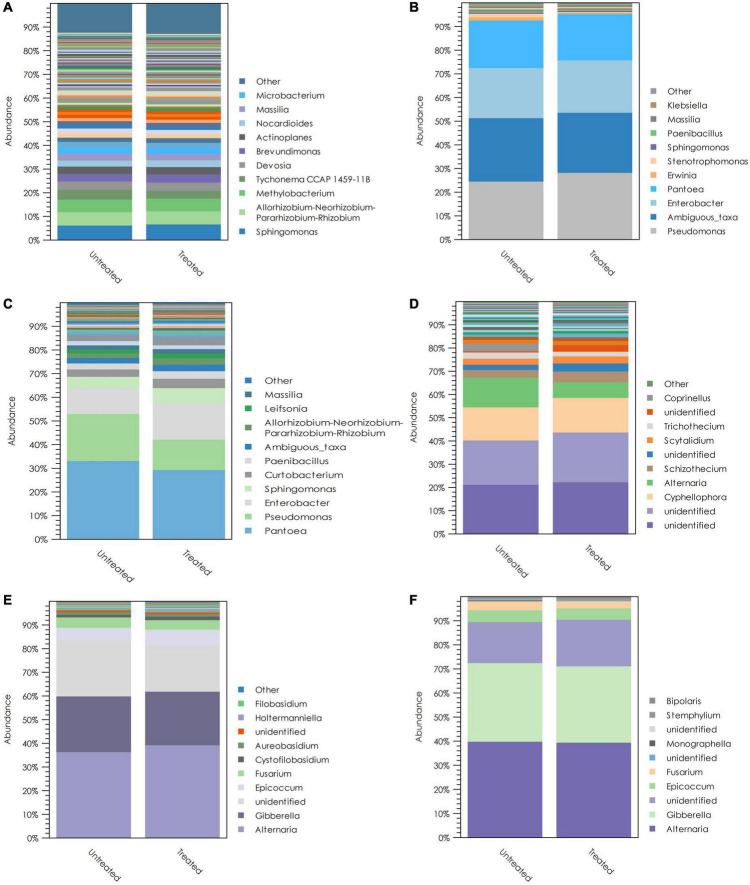
The dominant taxa and average relative abundances of bacterial taxa in **(A)** crop residue; **(B)** wheat spikes; **(C)** wheat kernels; and fungal taxa in **(D)** crop residue; **(E)** wheat spikes; **(F)** wheat kernels. Within each panel, the legend lists the ten most abundant genera, differentiated by color. Treatments have been grouped according to whether or not *T. gamsii* T6085 was applied to the tissue in question, and mean values are shown.

The *ITS* and *16S rRNA* amplicon sequences that we observed were used to generate OTU tables, by which the relative abundances of different fungal and bacterial taxa could be evaluated. Crop residue showed a comparatively different microbial community composition than wheat spikes and kernels (Figure 7). Several bacterial genera were dominant in residue. These include *Sphingomonas, Allorhizobium-Neorhizobium-Pararhizobium-Rhizobium, Methylobacterium*, and *Tychonema*. The two most abundant fungi in the residue were unidentified taxa within the phylum Ascomycota. The highest abundance taxa belonged to the class Dothideomycetes and order Pleosporales. However, the second most abundant taxon was only identified up to the phylum level. In addition to these two taxa, *Cyphellophora* and *Alternaria* showed higher relative abundances in crop residue. In addition, *Pseudomonas*, *Enterobacter*, and *Pantoea* were the highly dominant bacterial genera in wheat spikes and kernels. These genera represented more than 60% of the bacterial *16S rRNA* sequences observed. *Alternaria* and *Fusarium* were the predominant fungal genera in wheat spikes, and these two fungal genera accounted for more than 60% of the observed fungal *ITS* sequences from wheat spikes. Similarly, wheat kernels were highly dominated by *Alternaria* and *Fusarium*, which together accounted for more than 70% of the observed fungal sequences from wheat kernels (Figure 7).

## Discussion

The field experiment discovered a significant reduction in FHB incidence and severity due to the T6085 application (Figures 1, 2). A reduction of FHB DS in response to T6085 application to wheat spikes has been observed previously (Sarrocco et al., 2013). Anthesis is the most favorable period for *F. graminearum* to infect the wheat spikes. In addition, *T. gamsii* can endophytically colonize the wheat spikes, and this characteristic of T6085 is important to its potential as a biological control agent. However, over time, the beneficial effect of T6085 in FHB reduction was diminished. Therefore, a reduction in disease was not observed at the T_*sp*_3 time point. This fading of efficacy was expected both due to climatic conditions becoming particularly favorable to the development of the disease, and as T6085 was applied as an aqueous conidial suspension at a single time-point. The efficacy of biopesticides is strongly affected by conidial formulation and numbers, and application timing. Nevertheless, our observation of a significant reduction of symptoms on spikes at the T_*sp*_2 time point confirms previous observations.

The abundance of *F. graminearum* in crop residue and wheat spikes changed significantly throughout the period during which data were collected (Figure 3). In residue, *F. graminearum* was most abundant at the on-set of the experiment, and its density decreased over time. Although *Fusarium* spp. grow well in nutrient-rich residue and use residues from wheat, maize or rape for survival and reproduction, the pathogen is a poor competitor among the crop residue associated microbiome. For example, according to Leplat et al. (2016), *F. graminearum* growth rate is higher within sterile soil than in non-sterilized soil. In the presence of an antagonist, the abundance of *F. graminearum* frequently decreases over time (Leplat et al., 2013). In addition, the weather can also influence *Fusarium* abundance in crop residues over time. For instance, the perithecial development rate is lower during dry weather conditions (Manstretta and Rossi, 2016). Therefore, the amount of *F. graminearum* present in soil can also vary with the humidity. However, the *F. graminearum* abundance in wheat spikes showed an opposite trend, where at the initial time points, the *F. graminearum* abundance was significantly lower in wheat spikes, and it rose significantly at times closer to harvest. In other words, *F. graminearum* growth and development were enhanced during the seed maturation. This could be a natural outcome of having more time for *F. graminearum* to proliferate during the maturation of wheat spikes, and suggests that a second application of a biocontrol agent after anthesis could help reduce the further spread of the pathogen on the spikelets.

During the field study, two methods were used to inoculate the T6085 into crop residue and wheat spikes to obtain efficient colonization of the fungi to sufficiently mitigate *F. graminearum*. The crop residue was inoculated using sterilized millet seeds inoculated with T6085 spores and these millet seeds were hand distributed throughout the wheat field. For wheat spikes inoculation, a T6085 spore suspension was used as it allowed for a uniform distribution of the fungal spores throughout the wheat head. However, the T6085 treatment had a minimal impact on the abundance of *F. graminearum* in crop residue, wheat spikes and kernels (Figure 4). The method used to inoculate T6085 in crop residue and wheat spikes may not have been sufficient to establish the fungus in wheat tissues. The amount of millet seeds spread within the field may not enough, or *T. gamsii* T6085 may have colonized different portions of wheat spikes compared to *F. graminearum*. Non-uniform colonization of *T. gamsii* T6085 in wheat spikes was observed in an earlier field experiment using the same biocontrol agent in the 2011 and 2012 growing seasons (Sarrocco et al., 2013). In that earlier experiment, *T. gamsii* T6085 colonized lower spikelets more efficiently than upper spikelets. Non-uniform colonization of wheat spikes by the fungus could be due to the downward movement of spores toward the bottom of the spike with gravity when applying it as a spore suspension. Modifications that could be incorporated to enhance the establishment of *T. gamsii* T6085 could include applying a higher amount of fungal inoculum onto crop residue, increasing the spore concentration within the inoculum, adding an adhesive compound to the spore suspension to promote retention on the plant tissue, and spraying the inoculum with greater uniformly throughout the spikes (Sarrocco et al., 2013).

Moreover, weather conditions also play an essential role. If the weather conditions are more favorable (heavy and frequent rainfalls during the flowering and grain filling period—in April and May in Italy) for the pathogen over the biocontrol agent, the biocontrol agent may not achieve the density required to inhibit the pathogen. In the present experiment, rainfall was 140 and 100 mm during April and May 2019, respectively, much higher than the respective 30 year average, i.e., 75 mm in April and 62 mm in May (Supplementary Figure 2). During a similar field experiment in the 2011 growing season (21 and 8 mm rainfall in April and May, respectively), a significant reduction in FHB DS (% of affected spikelets within a spike) was observed when *T. gamsii* T6085 was applied on wheat spikes at anthesis. However, when the same field experiment was performed in the following year (160 and 60 mm rainfall in April and May, respectively, comparable to rainfall occurred in 2019) a significant reduction in DS was not observed (Sarrocco et al., 2013). Therefore, the efficacy of biocontrol by T6085 under field conditions can depend on multiple variables, not least the weather conditions during the growing season.

The changes in the microbiome in response to the introduction of the biocontrol agent *T. gamsii* T6085 as a biocontrol agent was evaluated to understand off target effects of the T6085 treatment on the wheatfield. The number of observed taxa (OTU richness) and the Shannon diversity index are commonly used estimates to measure the biodiversity of microbial communities and it is crucial to calculate the diversity indices to see the alteration made by the T6085 (Spellerberg and Fedor, 2003; Brown et al., 2007). In this study, the impacts of T6085 on OTU richness and Shannon index of the microbial community associated with crop residue, wheat spikes, and kernels were minimal. Yet, OTU richness and Shannon index variation over time were evident (Table 4), which demonstrates a minimal impact on the microbial community scale differences by the T6085 treatment.

Observing patterns and relationships among samples based on multivariate characterization, such as a profile of taxon presence and abundance, frequently requires the use of techniques to reduce the dimensionality of the data. We combined pairwise dissimilarity scores with ordination techniques to aid in visualization and to compare the microbial community composition among treatments and time points. The results suggested that there were not any community-scale impacts when T6085 was applied as a biocontrol agent (Figure 5). Nevertheless, some fungal and bacterial taxa were differentially abundant in T6085 treated samples compared to control samples; these may represent taxa that interact most directly with T6085. Yet, the impact of plant tissue type and the plant development stage was more apparent than the impact of T6085 colonization on the variations in microbial community structure (Figure 6). Similar results were observed in several other studies when biocontrol agents were applied for the prevention of other pathogens, including *R. solani* (Grosch et al., 2006; Kröber et al., 2014), *Phytophthora capsici* (Cucu et al., 2020), and *Verticillium dahliae* (Scherwinski et al., 2007). Compared to the experimentally imposed treatments, these studies have also encountered a more significant effect on microbial communities in response to the plant growth stage or time progression. Environmental changes other than maturation or development of the host plant tissue are also likely to impact microbial community dynamics (Lauber et al., 2013; Massart et al., 2015; Araujo et al., 2019; Latz et al., 2021).

The fungi and bacteria associated with different plant tissues vary among the type of tissue. In addition, the identity of the dominant fungal and bacterial taxa can similarly differ among plant tissues. Therefore, it is interesting to see the changes in the microbiome according to the niche in which they reside. Our results showed that in the year following their growth, wheat residues remaining in the field were primarily dominated by two unidentified *Ascomycota*, *Alternaria, Cyphellophora* and several bacterial genera with higher diversity. *Alternaria* and *Fusarium* were the dominant fungal genera in spikes and kernels from the current year’s growth, while *Pseudomonas*, *Enterobacter*, and *Pantoea* were the predominant bacterial species (Figure 7). The dominancy of *Fusarium* and *Alternaria* in wheat phyllosphere has been previously observed in several studies (González et al., 2008; Hertz et al., 2016; Schiro et al., 2018). According to Hertz et al. (2016), the dominant pathogen in a given location may depend on the climatic conditions. In the same study, they made only sparse observations of *F. graminearum* in wheat due to the dry climatic conditions during anthesis. Both *Fusarium* and *Alternaria* are pathogenic to wheat plants and contaminate the seeds with toxic compounds harmful to consumers. The presence of the two primary pathogens may have reduced the colonization of other fungi in the wheat plant through competition (Müller et al., 2018; Hoffmann et al., 2021). However, the predominant bacterial species, including *Pseudomonas*, *Enterobacter* and *Pantoea*, have overcome the competition from the two fungal pathogens and have well colonized the wheat spikes and kernels.

Interestingly, all these predominant bacterial genera include plant-beneficial and potential biocontrol agents (Chen et al., 2017; Kumar et al., 2017; Müller et al., 2018). According to Müller et al. (2018), *Pseudomonas* species have shown potential biocontrol ability against *F. graminearum*. Yet, successful development of FHB even in the presence of naturally occurring *Pseudomonas* spp. suggests that at natural population densities, these bacteria may not effectively inhibit FHB. Artificial inoculation at higher densities or combining with another biocontrol agent like *T. gamsii*, may increase the likelihood that these organisms could mitigate *F. graminearum* infection in wheat. Bakker and McCormick (2019) also observed similar bacterial and fungal compositions in wheat kernels from Minnesota, United States. Their study showed more than 100 bacterial taxa and several dozen fungal taxa in an individual wheat kernel and more significant microbial composition changes over the years. They found a negative correlation between *Fusarium* load and the relative abundance of *Sphingomonas* in wheat kernels. Our results also showed a higher relative abundance of *Sphingomonas* in wheat kernels. Therefore, the genus *Sphingomonas* could be explored for potential biocontrol agents to fight against FHB in wheat (Bakker and McCormick, 2019).

One drawback we encountered in this study was that we did not identify *Trichoderma* sequences within the microbiome profiles by the workflow we used; given that T6085 was applied to the plots, we expected to observe this taxon in our microbiome profiles. The mock community control samples we used included *Trichoderma reesei*, but we found that the *ITS2* sequences belonging to *T. reesei* could not be classified to narrower ranks than the family Hypocreaceae (data not shown). In observations from our biological samples, we observed OTUs that similarly could not be placed below Hypocreaceae; therefore, it is likely that *T. gamsii* was represented among these observations. Therefore, the method we used for fungal community profiling does not provide good discrimination among genera or species within this family. In addition, even though the *ITS* is considered a superior marker in species identification across the Kingdom fungi, the discriminatory power of *ITS* in Ascomycota is lower (Schoch et al., 2012).

## Conclusion

Our results indicated the critical role of the plant development stage, tissue type, and environmental conditions in maintaining the microbial community structure. However, the application of T6085 has a limited impact on the microbial populations associated with wheat plants, which suggests that the off-target effects of the use of T6085 as a biocontrol agent are not a concern.

## Data availability statement

The datasets presented in this study can be found in online repositories. The names of the repository/repositories and accession number(s) can be found below: https://www.ncbi.nlm.nih.gov/, PRJNA790613.

## Author contributions

SS, AB, GV, and MM designed and implemented the field experiment. AA performed the microbiome profiling and wrote the first draft of the manuscript. AB and AA worked in the portions of this work. All authors edited the manuscript and approved the final draft.

## Supplementary material

Supplementary Figure 1 Networks of co-associated sequence variants, for **(A)**. wheat spikes that were only mock inoculated, and **(B)**. wheat spikes that were inoculated with biocontrol agent *Trichoderma gamsii* strain T6085.

Supplementary Figure 2 The weather data during 2018/2019 growing season. The bar graphs show the rainfall and the line graph shows the maximal (red) and the minimal (green) temperature from July 2018 to June 2019.

Supplementary Table 1 List of sequence variants that were significantly differentially abundant (DEseq2, adjusted *p* < 0.05) between samples that were untreated vs. treated with T6085.

Supplementary Table 2 Lists of bacterial sequence variants that were significantly differentially abundant (DEseq2, adjusted *p* < 0.05) between sampling time points within a given tissue type.

Supplementary Table 3 Lists of fungal sequence variants that were significantly differentially abundant (DEseq2, adjusted *p* < 0.05) between sampling time points within a given tissue type.
