# Supplementary figures and images for: Minimal impacts on the wheat microbiome when Trichoderma gamsii T6085 is applied as a biocontrol agent to manage fusarium head blight disease
Source: Front Microbiol. 2022 Sep 23;13:972016. doi: 10.3389/fmicb.2022.972016 (PMC9539683; doi:10.3389/fmicb.2022.972016)

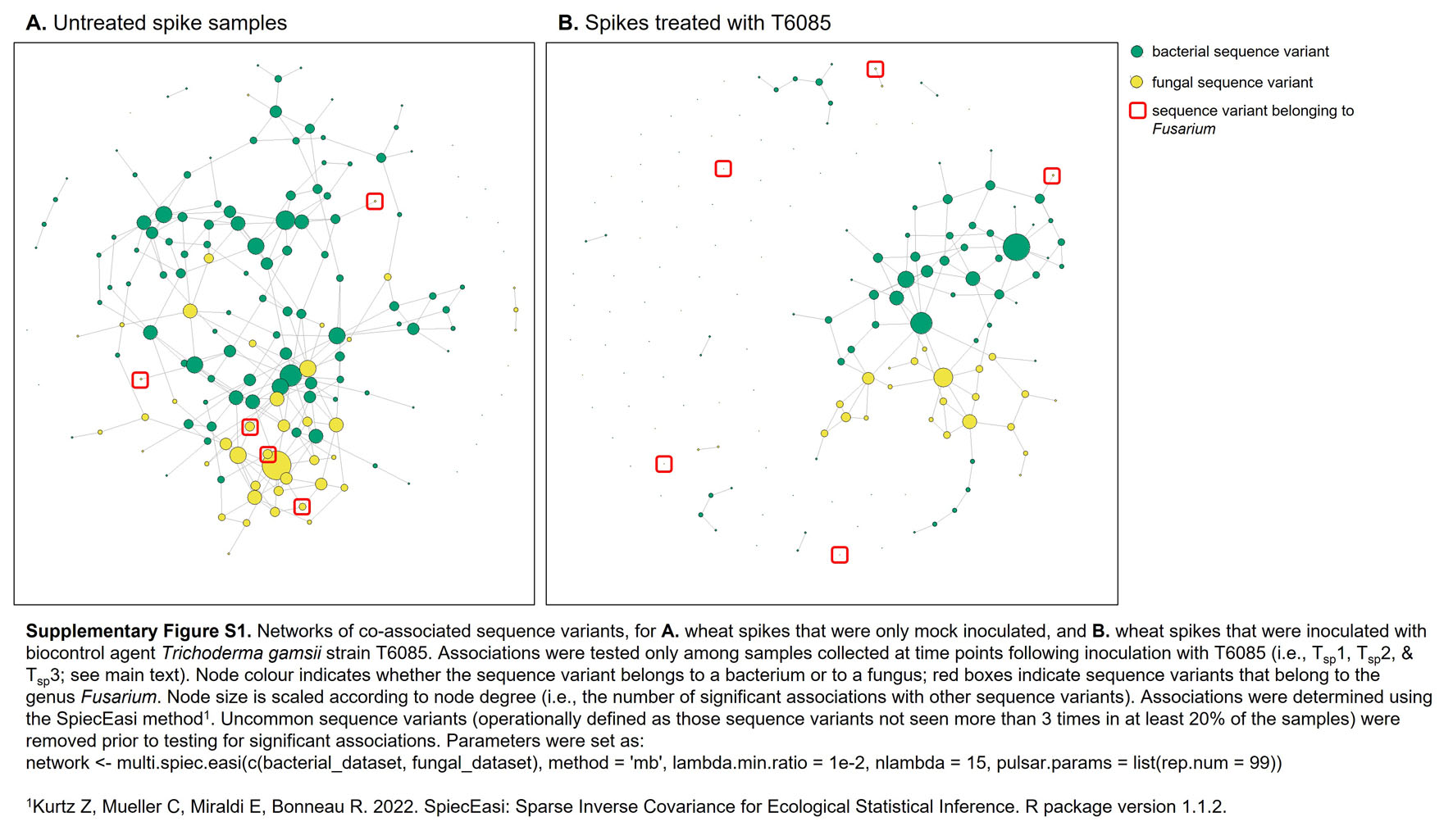

Supplement: Supplementary Figure 1 — Networks of co-associated sequence variants, for (A). wheat spikes that were only mock inoculated, and (B). wheat spikes that were inoculated with biocontrol agent Trichoderma gamsii strain T6085. [file Image_1.jpeg]

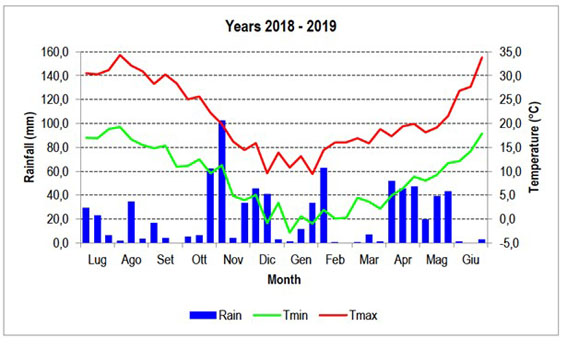

Supplement: Supplementary Figure 2 — The weather data during 2018/2019 growing season. The bar graphs show the rainfall and the line graph shows the maximal (red) and the minimal (green) temperature from July 2018 to June 2019. [file Image_2.jpeg]
